# A Case of Hydrocele, Reported to the Medical Society of Tennessee at Its Annual Meeting in May

**Published:** 1841-08

**Authors:** Ferdinand Smith

**Affiliations:** Franklin, Tenn.; Nashville


*Nashville, May* 3, 1841.

---

Art. V.—*A Case of Hydrocele,* reported to the Medical Society of Tennessee at its meeting in May. By Ferdinand Stith, M. D., of Franklin, Tenn.

A young gentleman from the country, aged about 25 years, of fair skin, light hair, and blue eyes, called on me, on the 17th of March, 1836, to have himself relieved of a disease of the testis. On examination, I found him the subject of hydrocele, of two or three years standing. I explained to him the nature of his case, and gave him to understand what sort of an operation he would have to submit to, and how long he would probably be confined.

He concluded to undergo the operation at once, and accordingly took lodgings in town for that purpose. He manifested great solicitude to keep the nature of his disease secret, and desired me, if I could, to dispense with the aid of an assistant, to which I readily consented; and having made the necessary preparations, I repaired to his room for the purpose of operating.

Having placed him in a favorable position on the edge of the bed, and making myself acquainted with the position of the testis, I laid open the sac with my scalpel to the extent of an inch and a half. So soon as the encysted fluid, amounting to about a pint, had escaped, I introduced my finger into the tunica vaginalis testis, for the purpose of bringing its surface, and the surface of the testis, into view, in order that I might the more correctly judge of the condition of the parts. I found the surface generally presenting a normal appearance, all but a portion directly over the testis, on which there was a slight roughness to the touch, the sensibility of which was so exquisite, with the excessive nervousness of the patient, that when I attempted to introduce a fold of the softest lint, I found him falling into syncope, and was compelled to desist.

After giving him some stimuli and waiting a few minutes, I made a second attempt to introduce a fold of lint, made still less irritating by covering it with simple cerate. This time I succeeded partially, but the operation gave him great pain. I passed a small strip of lint above the testis, in the direction of the spermatic cord. I had made up my mind to effect a radical cure, and had so promised my patient; but at this stage of the business I began to feel in some doubt as to the best mode of proceeding. I was afraid of the effects of stimulating injections on a surface that would not bear the slightest touch, but could not think of suffering the incision to heal without obliterating the cavity of the sac. And it now occurred to me, that the least irritating article that could be devised, would be a piece of the softest kid skin, such as I use for animal ligatures, saturated with the mucilage of gum arabic and white sugar. This I succeeded in introducing with a probe, without giving the slightest pain.

The strips of lint and of kid skin having now been introduced, in different directions, I proceeded to close the wound, leaving the ends of the strips slightly projecting from its lips, and applied a suspensory bandage.

In the course of two or three days the adhesive inflammation was fully established—indeed, was something more than I had desired, and I had to bleed him once, and give him two or three gentle purgatives. In the course of thirty-six or forty-eight hours from the time of the operation, the kid skin had become fixed in its position by the adhesive inflammation, and on the sixth day the projecting extremities dropped off, that portion of the sac in which it lay having contracted and firmly united. While this was the case with one portion of the sac, that which was occupied by the lint had not suppurated sufficiently to allow me to withdraw it, until the ninth day. On the twelfth day, all the external openings had closed, and I put the patient on the use of camphorated mercurial ointment to hasten the reduction of the parts to their natural condition. He was now able to leave his room, had begun to walk about town, and after a few days more, returned in good health to the country. He has never been threatened with a return of the symptoms, and is now, as he has been at all times since he recovered from the operation, in the enjoyment of fine health.

My object in reporting this case to the Society is two-fold; first, to direct the attention of surgeons to the use of the kid skin in such cases, as giving less pain, and being more likely to answer every indication than any agent hitherto employed;—and, secondly, to suggest the idea of attempting the radical cure of hernia, by, in the first place, reducing it, then opening the sac, and, lastly, introducing the kid skin. Of course, for some days, the patient, after such an operation, would be obliged to maintain the horizontal posture, to use compresses so adjusted as to prevent the intestine from falling into the passage, and to keep the parts in a state of absolute rest. The union, in my opinion, would be effected in forty-eight hours; and if the patient were in a favorable state for the operation, and received judicious treatment afterwards, I should not apprehend any danger of an extension of the inflammation beyond the limits of the enclosed kid skin.

